# Expression and activity of Rac1 is negatively affected in the dehydroepiandrosterone induced polycystic ovary of mouse

**DOI:** 10.1186/1757-2215-7-32

**Published:** 2014-03-14

**Authors:** Vineet Kumar Maurya, Chadchan Sangappa, Vijay Kumar, Sahil Mahfooz, Archana Singh, Singh Rajender, Rajesh Kumar Jha

**Affiliations:** 1 Division of Endocrinology, Life Science North 111B/101, CSIR-Central Drug Research Institute, B.S. 10/1, Sector-10, Jankipuram Extension, Sitapur Road, Lucknow 226031, India; 2 Division of Endocrinology, Life Science South, CSIR-Central Drug Research Institute, B.S. 10/1, Sector-10, Jankipuram Extension, Sitapur Road, Lucknow 226031, India

**Keywords:** Polycystic ovary, Dehydroepiandrosterone, Rac1, Estradiol, Vav, Caveolin1

## Abstract

**Background:**

Polycystic ovarian syndrome (PCOS) is characterized by the presence of multiple follicular cysts, giving rise to infertility due to anovulation. This syndrome affects about 10% of women, worldwide. The exact molecular mechanism leading to PCOS remains obscure. RhoGTPase has been associated with oogenesis, but its role in PCOS remains unexplored. Therefore, we attempted to elucidate the Vav-Rac1 signaling in PCOS mice model.

**Methods:**

We generated a PCOS mice model by injecting dehydroepiandrosterone (DHEA) for a period of 20 days. The expression levels of Rac1, pRac1, Vav, pVav and Caveolin1 were analyzed by employing immuno-blotting and densitometry. The association between Vav and Rac1 proteins were studied by immuno-precipitation. Furthermore, we analyzed the activity of Rac1 and levels of inhibin B and 17β-estradiol in ovary using biochemical assays.

**Results:**

The presence of multiple follicular cysts in ovary were confirmed by histology. The activity of Rac1 (GTP bound state) was significantly reduced in the PCOS ovary. Similarly, the expression levels of Rac1 and its phosphorylated form (pRac1) were decreased in PCOS in comparison to the sham ovary. The expression level and activity (phosphorylated form) of guanine nucleotide exchanger of Rac1, Vav, was moderately down-regulated. We observed comparatively increased expressions of Caveolin1, 17β-estradiol, and inhibin B in the polycystic ovary.

**Conclusion:**

We conclude that hyperandrogenization (PCOS) by DHEA diminishes ovarian Rac1 and Vav expression and activity along with an increase in expression of Caveolin1. This is accompanied by an increase in the intra-ovarian level of '17 β-estradiol and inhibin B.

## Introduction

The pool of primordial follicles in the ovary supply eggs for the entire reproductive life in mammals. To maintain fertility for the whole reproductive period, the primordial follicles are reserved in a quiescent state for regulated successive ovulation [1-3]. Primordial follicles are recruited from the reserve of dormant follicles into the pool of growing follicles through their activation process during which they undergo a series of developments [3]. Polycystic ovarian syndrome (PCOS) is characterized by anovulation and in the presence of multiple small cysts typically arranged in the periphery of one or both ovaries. PCOS can affect 5–10% of women during their reproductive age and contributes to this etiology in about 10% of the infertile women [4-6]. This disorder is considered to be a manifestation of the disturbance in the endocrine system, which causes secondary disorders contributing to female infertility [7]. The most commonly seen endocrine disturbance is hyperandrogenism accompanied by chronic oligo or anovulation [8]. The hypothalamic-pituitary synchrony is disrupted that increases pulsatile secretion of gonadotropin, disturbs oocyte-granulosa cell interaction, enhances ovarian androgen production and causes excess insulin production and that leads to insulin resistance [9]. As a result, metabolic syndrome (MS) is seen in about 46% of the PCOS cases [10].

However, the intra-ovarian pathophysiology of PCOS is not yet explicit at cellular and molecular levels. The available data till date is insufficient to precisely delineate the intra-ovarian pathway that contributes to the development of this disorder. Therefore, we need further investigations to pinpoint the correct mechanism leading to the development of this disorder. The G-protein family member, Ras, has already been shown to participate in the pathophysiology of PCOS [11]. Another member of Rho family protein, Rac, is involved in gonad formation [12] and acts downstream to integrin signaling [13]. Its expression/activity is controlled by estrogen [14]. Rho guanine dissociation inhibitor (RhoGDI) antagonizes Rac1 and keeps it in the inactive state [15,16]. The Rho assists in actin dynamics through cofilin regulation by Luteinizing hormone signaling [17] in the granulosa cells [18]. Furthermore, Rac modulates cell cycle [19], which is activated by guanine exchange factor Vav [20,21]. Further, it is reported that Rac gets phosphorylated in the process of its activation [22]. Similarly, Vav also gets phosphorylated before it executes Rac activation [23,24]. Looking at the role of Rac/Vav signaling in ovarian physiology, we designed the present study to analyze expression and activity of Rac1 and Vav proteins in the ovary of a mice model of PCOS.

## Materials and methods

### Reagents

Dehydroisoandrosterone 3′-sulphate (cat no. D5297), hematoxylin (cat no. H3136), anti-beta-actin (cat no. A2668) and goat anti-Mouse IgG (γ-chain specific)-HRP (cat no. A3673) were purchased from Sigma Aldrich Inc., St Louis, MO, USA. Immobilon-P PVDF membrane (0.45 μm), ECL reagent kits (cat no. WBKLS0500), Protein-A-Agarose suspension (cat no. IP02) and goat anti-rabbit-HRP IgG (cat no.621140380011730) were procured from Merck-Millipore, Cedex, France. Other primary antibodies against phospho-Vav (Y174) (cat no. ab47282), phospho-Rac1/Cdc42 (S71) (cat no. ab5482) and Rac1 (cat no. ab33186) were purchased from Abcam, Cambridge, MA, USA. Anti-Vav (cat no. sc132) and anti-Caveolin1 (cat no. sc894) were purchased from Santa Cruz Biotechnology, CA, USA. Non-fat milk (cat no.170-6404) and precision plus protein standard marker (cat no. 161-0374) were obtained from Bio-Rad Lab., Inc., Hercules, CA., USA. Protein assay kit (cat no. 23225) was procured from Thermo-Scientific, Rockford, USA. The G-LISA Rac1 activation assay Biochem Kit (cat no. BK128) was purchased from Cytoskeleton, Denver, CO, USA. Inhibin B Enzyme Immunoassay kit (cat no. EIA-INB-1) was purchased from RayBiotech, Inc., Norcross, GA, USA. 17 β-estradiol assay kit (cat no. ADI-900-008) was obtained from Enzo Life Science, Inc., Farmingdale New York, USA.

### PCOS experimental animal model

The murine model of PCOS was developed by administering *Mus musculus* (strain C57/BL6) with dehydroisoandrosterone (DHEA). PCOS was induced in 22 days old mice (12 gm) by injecting DHEA (6 mg/100gm body weight; dissolved in 0.01 ml 95% ethanol, which was further diluted with corn oil) subcutaneously for 20 consecutive days as described previously [25-28]. The model is characterized by higher levels of serum testosterone, androstenedione and 5-alpha-dihydrotestosterone similar to that seen in PCOS patients. Previous studies have established that the DHEA-PCOS murine model represents some of the salient features of human PCOS, such as hyperandrogenism, abnormal maturation of ovarian follicles and anovulation [26,27,29-31]. We administered corn oil along with 95% ethanol in the control (sham) group. Animal usage and the protocols were duly approved by the Institutional Animal Ethics Committee of the CDRI, Lucknow, India. The animals were housed in a temperature-controlled facility (25 ± 1°C) with required illumination (12 h light and 12 h dark). Free access to food and water were provided to the animals. At the end of the experiments, animals were sacrificed by cervical dislocation followed by excision of ovaries, which were snap frozen at −80°C until further use. Each treatment/control group consisted of six animals.

### Ovarian tissue histology and staining

To evaluate the histological alteration in the ovary, DHEA treated and sham/control ovaries were dissected and allowed to fix overnight at 4°C in 4% paraformaldehyde (PFA)-phosphate buffered saline (PBS). On subsequent day, tissue samples were kept in the tissue cassette and dehydrated using acetone (two times for 30 min each), acetone + benzene (1:1, 30 min) and cleared in benzene (two times for 30 min each). Subsequently, the tissues were removed and embedded in paraffin wax (Fisher Scientific, Rockford, USA) for 4 h at 65°C. This was followed by preparation of tissue paraffin moulds.

Embedded ovarian tissues were sectioned (5 μm) using microtome (Leica Biosystem, Germany) and mounted on poly-L-lysine (Sigma-Aldrich, MO, USA) coated glass slides. Sections were deparaffinized with two changes of xylene (10 min each) and rehydrated with subsequent changes of absolute alcohol (two times, five min each), 95% (two min) and 70% alcohol (two min). Sections were briefly washed (three times) and stained with hematoxylin solution for eight min. After staining, sections were again washed and kept for blue color development in 1.5% ammonium hydroxide (30% stock) for 30 sec. The tissue section mounted slides were washed in distilled water for five min, rinsed in 95% alcohol (10 repeats) and counterstained with 0.5% eosin for 30 sec. This was followed by dehydration through 95% and absolute alcohol two times for five min each. Finally, the slides were cleared in xylene two times for five min each and mounted with DPX mountant. The tissue sections were imaged through Inverted Phase Contrast Microscope (TS100-F, Nikon, Japan) using 5.2 megapixels digital camera (DS-Fi2-U3, Nikon, Japan).

### Ovarian protein extract preparation

After excision, the ovaries were processed for total (cytosol + plasma membrane) protein extract preparation. The ovarian tissue was minced and homogenized in a buffer containing 100 mM KCl, 3 mM NaCl, 3.5 mM MgCl_2,_ 10 mM PIPES, 1.5 mM EGTA, 1 mM PMSF, 50 g/ml, phosphatase, and protease inhibitors (pH-7.4) [32]. The tissue homogenate was centrifuged at 200 × g for 10 min at 4°C to pellet-out unbroken cells and tissue debris. Later, the mitochondrial fraction was removed by centrifuging the preparation at 12,000 × g. The concentration of protein was estimated using Pierce BCA protein assay kit as per the manufacturer’s instructions. Suitable concentration (20μg) of protein extract was prepared for down-stream purposes.

### Rac1 activity assay

The activity of Rac1 (GTP bound form) was assayed in the ovarian protein extract using G-LISA Rac1 activation assay Biochem Kit, as per the manufacturer’s instructions and that is already validated [33]. Briefly, a total of 50 μg protein extract was added to each corresponding well pre-coated with Rac-GTP-binding protein. This was incubated at 4°C for 30 min followed by successive incubation with 50 μl of anti-Rac1 for 45 min. Later, secondary antibody conjugated with HRP (50 μl) was incubated for 45 minutes. Subsequently, 50 μl of HRP detection reagent was added to each well, followed by incubation for another 20 min. The reaction was stopped by the addition of 50 μl of HRP stop solution and the absorbance was recorded at 490 nm.

### Inhibin B assay

The level of inhibin B was determined in the ovarian protein extract using RayBio Inhibin B Enzyme Immunoassay kit as per the manufacturer’s instructions. We added 100 μl of inhibin B antibody in each well of micro-plate, which was pre-coated with anti-rabbit antibody. The plate was incubated overnight at 4°C with gentle shaking. This was followed by the addition of 100 μl of protein sample to each well and incubation overnight at 4°C. HRP-Streptavidin was added followed by incubation at room temperature for 45 min. Subsequently, 100 μl of 5’-tetramethylbenzimide (TMB) was added as HRP detection reagent and incubated at room temperature in dark for 30 min. The reaction was terminated by adding 50 μl stop solution and absorbance was recorded at 450 nm.

### 17 β-estradiol hormone estimation

The assay was performed as per instructions provided by the manufacturer. A total of 40 μg (100 μl) ovarian protein (n = 4; sample pooled from 10 animals to form 4 replicate in each group) was used for this assay. The standard of 17 β-estradiol was prepared by the addition of 100 μl standards (30,000 pg/ml, 7,500 pg/ml, 1,875 pg/ml, 468.8 pg/ml, 117.2 pg/ml and 29.3 pg/ml) and assay buffer3 (50 μl) into respective standard wells, whereas the standard diluent (100 μl) was added to well B_0_ (maximum binding well). The NSB (negative control) received standard diluents (100 μl), assay buffer3 (50 μl) and conjugate (phosphatase conjugated with 17β-estradiol) (50 μl). Blank was prepared by adding only substrate (200 μl) and stop solution (50 μl). Next, we added 50 μl of conjugate in all the wells except blank. Antibody against 17β-estradiol (50 μl) was added in wells B_0_, standard, and samples only followed by incubation of plate at room-temperature (RT) for 2 h. The unbound/excess content was decanted and micro-plate was washed thrice with 400 μl of wash buffer by gentle tapping on paper towel. Thereafter, a substrate (p-nitrophenyl phosphate; 200 μl) was added in each well followed by incubation at RT for 45 min. Finally, a stop solution (trisodium phosphate; 50 μl) was added to each well and micro-plate was read at 405 nm using micro-plate reader (SPECTRO star Nano, BMG LABTECH, GmbH, Germany). The graph was plotted with absorbance versus standards (17β-estradiol; pg/ml) after subtraction of NSB values. The concentration of 17β-estradiol was calculated based on the standard curve.

### Co-immuno-precipitation assay

The ovarian protein lysate (100 μg, sham group) from each replicate was immuno-precipitated by incubation with anti-Vav overnight at 4°C. To collect the immune complexes, Protein-A-Agarose (20 μl) was added and the lysate-bead mixture was incubated at 4°C under rotary agitation for 4 h. It was followed by centrifugation at 10,000 × g for 10 min at 4°C and washing three times with PBS. Protein was eluted with Laemmli buffer and boiled for 5 min. The supernatant was subjected to 12% SDS-PAGE and immuno-blotted with anti-Rac1 according to a previously described method [32].

### SDS-PAGE and Western blotting

Protein sample (20 μg) was denatured by boiling in Laemmli buffer [34] for 5 min at 95°C and applied on a 10-12% SDS-PAGE. The proteins resolved on the gel were transferred to PVDF membrane (0.45 μm) in transfer buffer (20% methanol, vol/vol; 0.19 M glycine; 0.025 M Tris-Base, pH = 8.3) [35]. The membrane was blocked with 5% non-fat milk/goat serum and incubated overnight at 4°C with antibodies against Rac1 (1:1000 dilution), pRac1 (1:1000 dilution), Vav (1:250 dilution), pVav (1:1000 dilution), Caveolin1 (1:1000 dilution) and beta-actin (1:6000 dilution). Thereafter, the membranes were incubated for one h with goat anti-rabbit IgG or goat anti-mouse IgG conjugated with Horseradish Peroxidase in a paraffin boat. Phosphate buffered saline (10 mM, pH 7.4 containing 0.1% Tween-20, PBS-T) was used throughout the procedure. Later, the membranes were exposed to ECL reagents to visualize the protein bands and imaged through Chemi-Imager (ImageQuant LAS4000, Buckinghamshire, UK). Immuno-positive bands were analyzed by densitometry using Total Lab Quant 1D software (Nonlinear Dynamics Ltd., UK). The beta-actin blot values were used to normalize the blots value of Rac1, pRac1, Caveolin1, Vav and pVav.

### Statistical analysis

All the experiments were performed in three replicates using six animals in each group. Ovarian tissue samples were pooled from two animals to form one replicates to increase the yield of protein extract. Protein band intensities were averaged and the standard error of the mean (SE) was calculated. The data were subjected to one-way ANOVA using Microsoft Excel 2007. P values less than 0.05 were considered significant for statistical inference.

## Results

### Characterization of PCOS by histological analysis

**Figure 1:**
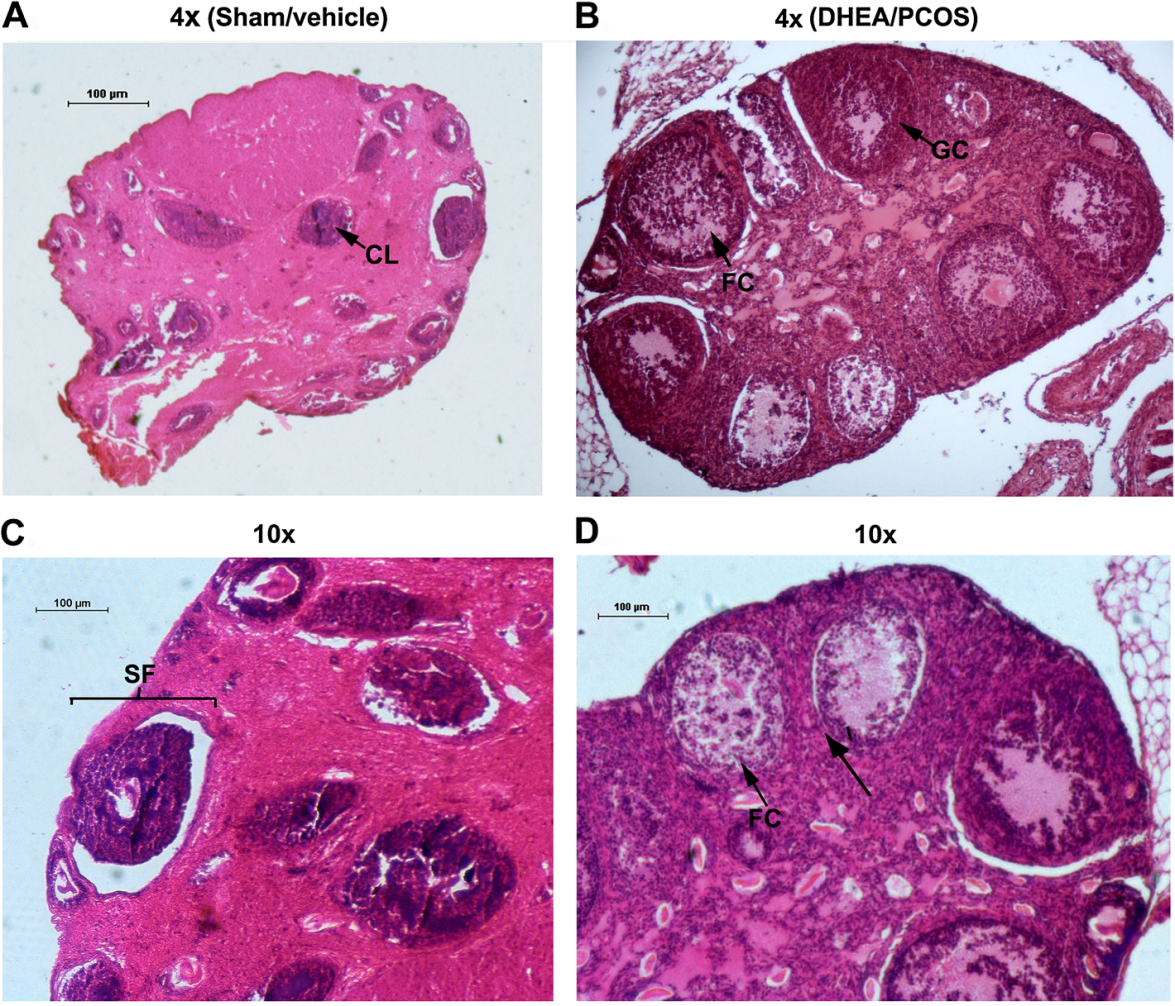
**Morphological comparison of DHEA treated mouse ovary with its control.** Sham/vehicle ovary showing (4x magnification) corpus luteum and secondary follicle **(A and C)**, DHEA treated PCOS ovary showing (10× magnification) follicular cyst (FC) along with degenerate granulosa cells **(B and D)**. Corpus-luteum was completely absent in polycystic ovary.

To demonstrate the effect of DHEA, ovaries were sectioned and stained with hematoxylin/eosin. Ovarian sections of control (sham) group showed the presence of follicles at different stages of maturation (Figure 1). Atretic, graffian and healthy follicles were clearly visible along with corpora lutea (Figure 1A). The presence of healthy oocytes in the follicles was seen (Figure 1C). In contrast, DHEA treated ovary exhibited a bit distorted morphology (Figure 1B). The numbers of antral and pre-natal follicles were increased with arrangement typically that of the polycystic ovary; however, the granulosa cells appeared to be degenerated (Figure 1D). The size of the polycystic ovary was increased, perhaps due to an increase in the number of follicles. Oocytes and corpora-lutea were not seen in the polycystic ovary.

### Increased expression level of inhibin B in polycystic ovary

**Figure 2:**
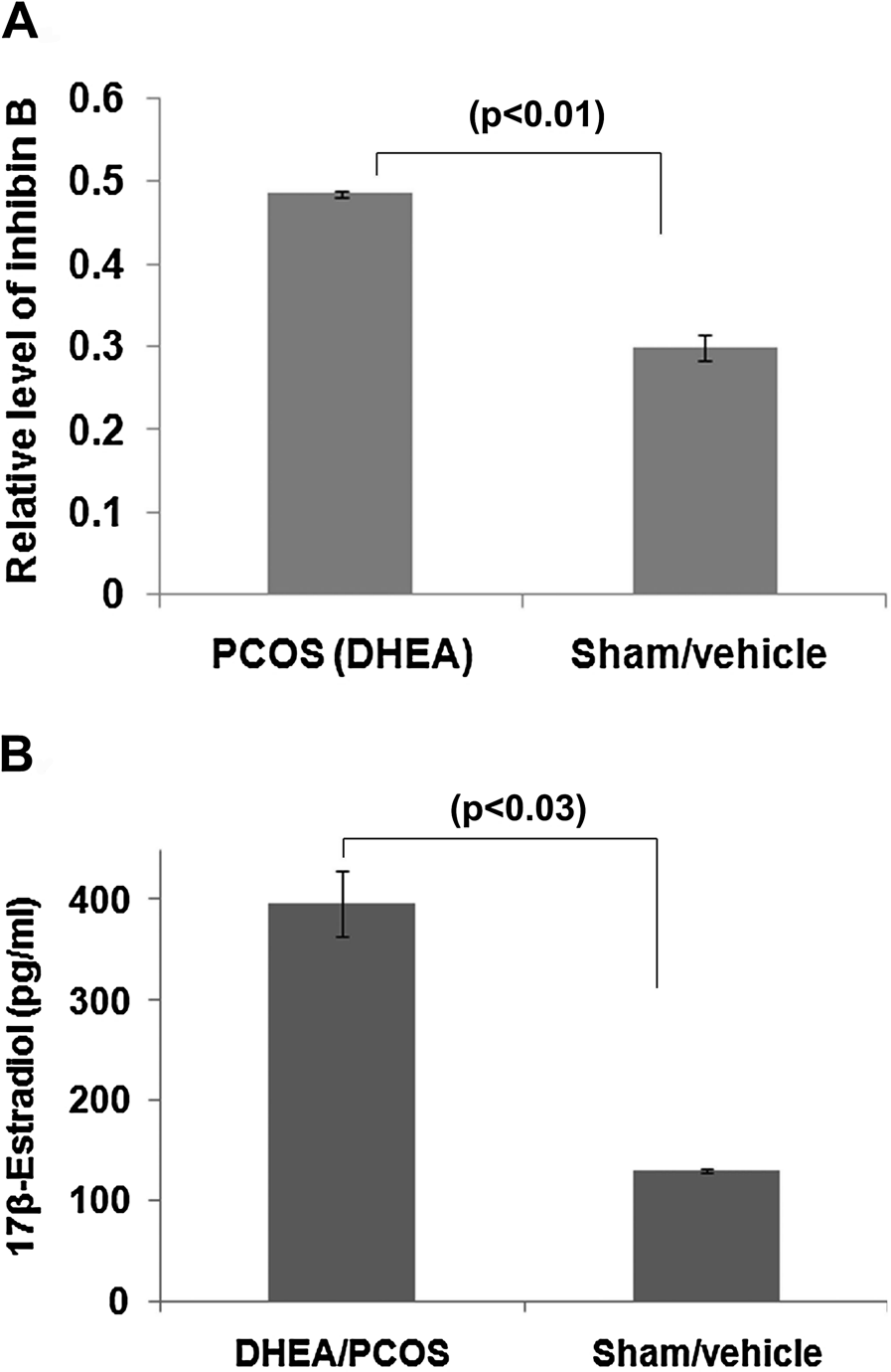
**Level of inhibin B and 17β-estradiol in sham and DHEA treated mouse ovary.** The intra-ovarian level of inhibin B **(A)** and 17-β-estradiol **(B)** were assessed post treatment of DHEA/sham in mouse.

The level of inhibin B correlates with the number of follicles recruited to undergo maturation [36]. Earlier studies have reported high concentrations of serum inhibin B in polycystic ovaries [37-39]. Herein, we performed assay of inhibin B in ovarian tissue protein extract to validate the pathophysiology of PCOS in our animal model. We observed a significant increase (p < 0.015) in level of inhibin B in the polycystic ovaries in comparison to sham (control) ovaries. Our result showed ~61% elevation in the level of inhibin B (Figure 2A).

### Elevated level of 17 β-estradiol in the PCOS ovary

Serum level of 17β-estradiol (E_2_) indicates the development of dominant follicles [36]. Therefore, we analyzed intra-ovarian level of 17β-estradiol in the PCOS and control groups. We observed about three folds elevation in the level of estradiol in PCOS ovary in comparison to the control ovary (p < 0.03) (Figure 2B).

### Down regulation of activity and expression of Rac1 in PCOS ovary

**Figure 3:**
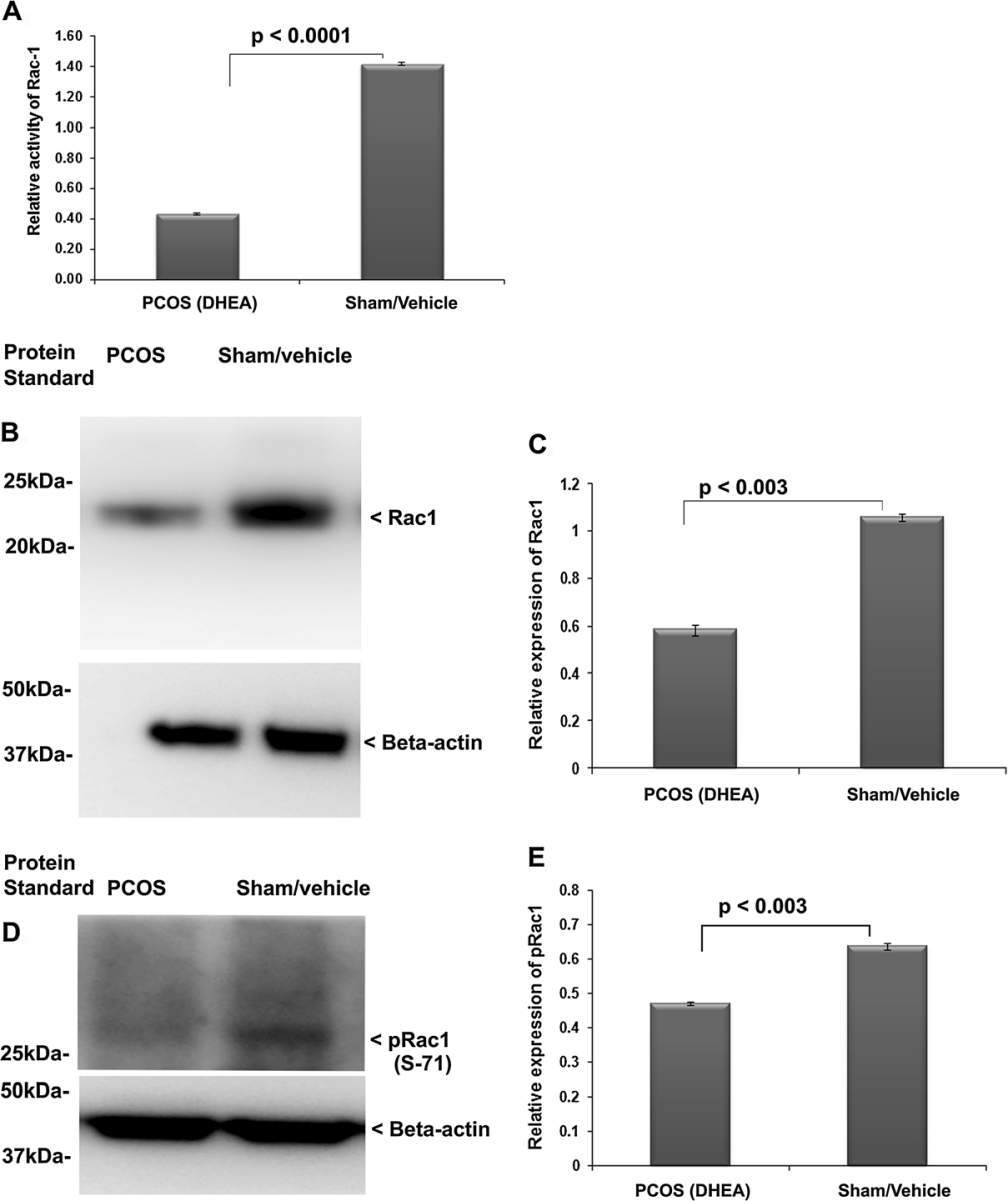
**Determination of expression and activity of Rac1 in DHEA induced PCOS ovary.** Relative activity of Rac1 was analyzed in the DHEA (PCOS) and sham treated ovary **(A)**. Expression level analysis of Rac1 was determined by immuno-blotting and densitometry in the ovary of PCOS and sham **(B and C)**. The activity Rac1was assayed by analysis of its phosphorylated form **(D and E)**.

In order to decipher the possible involvement of Rac1 signaling in PCOS, we detected the expression level of Rac1 and phosphorylated-Rac1 (S71) (pRac1) in the ovarian tissue protein extract prepared from the experimental model of PCOS. As shown in Figure 3A, compared with the sham/vehicle treated ovaries, the activity level of Rac1 was significantly decreased in the PCOS ovary. Similarly, the expression level of Rac1 protein was significantly down-regulated in DHEA treated/PCOS ovary (Figure 3B and C).

Further, to determine whether the activity of Rac1 in terms of its phosphorylation was affected in the DHEA treated (polycystic) ovary, the phosphorylation level of Rac1 was analyzed employing immuno-blotting. Treatment of ovary with DHEA resulted in down-regulation of pRac1 (Figure 3D and E). Thus, this domain of information suggests that DHEA/polycystic ovary may adversely affect the activity of Rac1.

### Association of Vav activity in PCOS ovary

**Figure 4:**
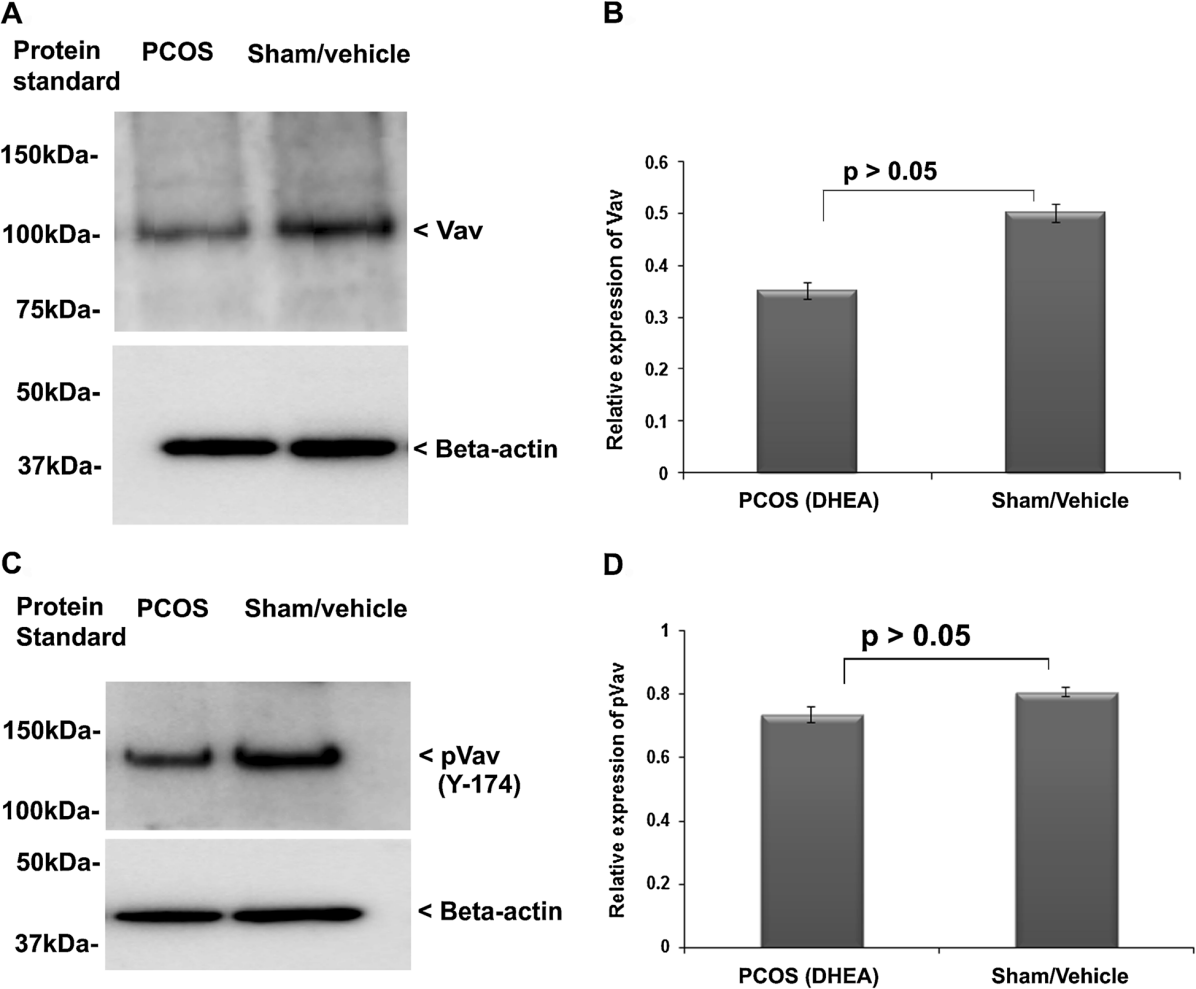
**Vav and pVav expression analysis in the PCOS and sham ovary.** Phosphorylated-Vav (pVav) was studied in the PCOS (DHEA treated) ovary **(A and B)** using Western blotting. Expression level of total Vav and pVav were analyzed using densitometry **(C and D)**.

Rac1 is activated by Vav, which gets phosphorylated at tyrosine (Y) 174 [40]. To evaluate whether DHEA can also modulate of intra-ovarian activity of Rac1, we further evaluated the activity and expression level of Vav in the ovary after DHEA treatment (polycystic ovary). The activity of Vav in terms of its phosphorylation was analyzed through phosphorylation assay/Western blots (Figure 4). We found reduced expression level of total Vav in the hyper-androgenized ovary, but the difference in comparison to control was not statistically significant (Figure 4A and B). Similarly, the intensity of anti-pVav (Y174) positive band was found to be lower in PCOS in comparison to the control (sham) group (p > 0.05) (Figure 4C and D).

### Vav can interact with Rac1 in ovary

**Figure 5:**
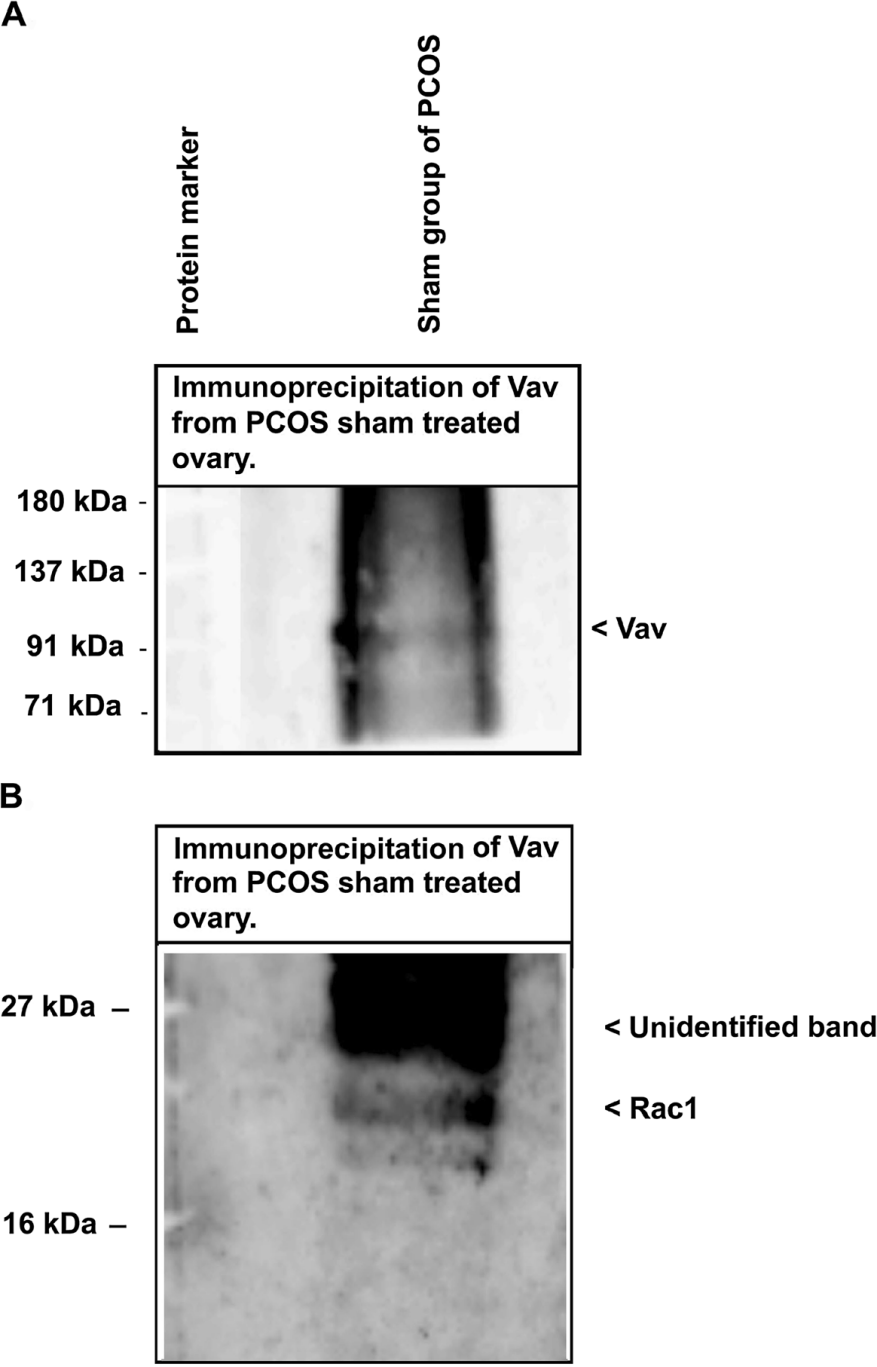
**Analysis of Rac1 and Vav interaction in the ovary.** Lysate from PCOS ovary was processed for Vav immuno-precipitation followed by immuno-blotting with Rac1 antibody **(A)**; presence of Rac1 in the Vav IP was confirmed by its immuno-blotting **(B)**.

It is well known that Vav displays guanidine exchange factor activity for Rho GTPases. We performed co-immuno-precipitation analysis to confirm the Rac1 association with Vav in the sham ovary. The Vav presence in the Vav antibody IP was confirmed by its immuno-blotting (Figure 5A). As shown in Figure 5B, the immuno-precipitated protein samples by Vav antibody from the ovary of sham administered displayed immuno-positive band of Rac1, which is an indication that Rac1 interacts with Vav in the ovary.

### Caveolin1 is up-regulated in PCOS ovary

**Figure 6:**
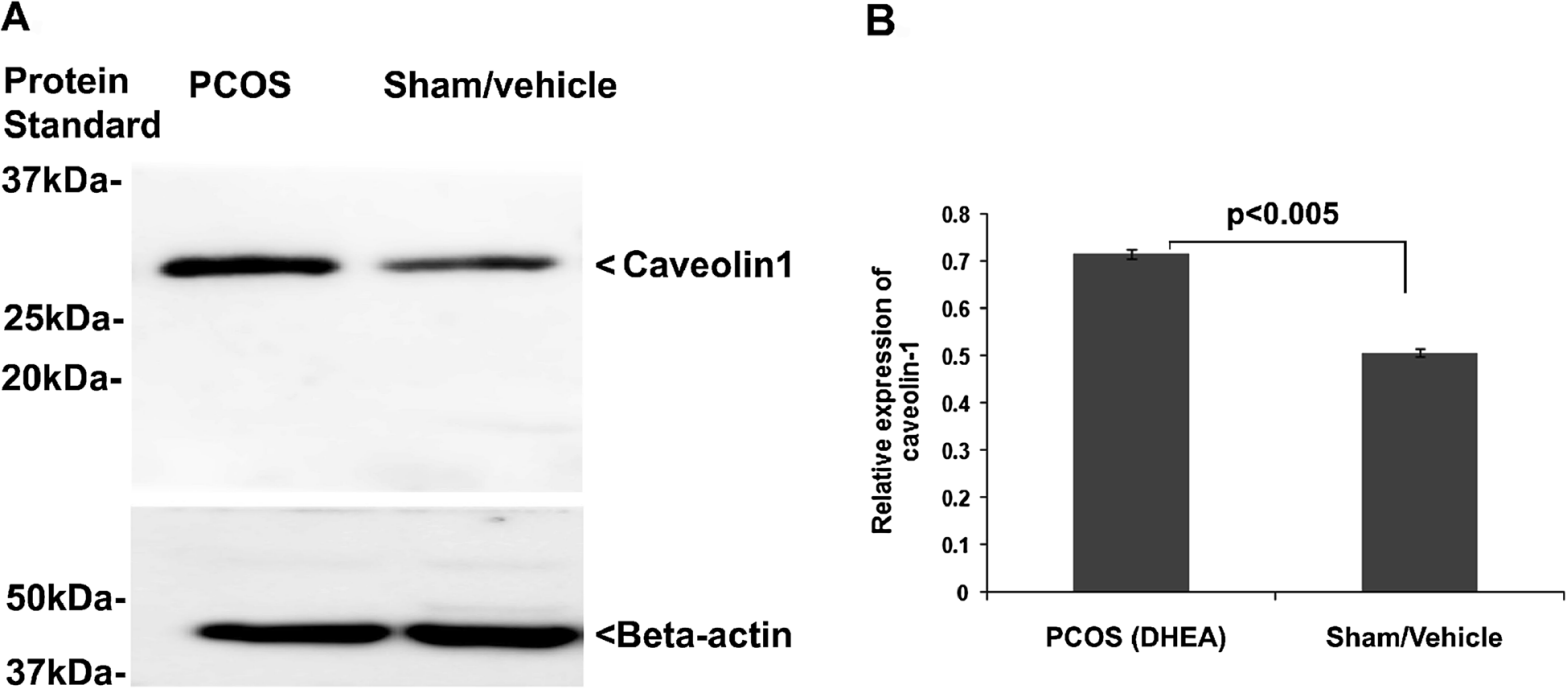
**Evaluation of expression of Caveolin1 in the DHEA and sham treated ovary.** Immuno-blotting of Caveolin1 was performed in PCOS and sham ovary group in comparison to the control group **(A)**; Densitometric analysis of Cav1 in PCOS and control group **(B)**.

Caveolin1 is known to be an important scaffolding domain containing caveolae protein, which is involved in the regulation of several signaling cascades [41]. Since, Caveolin1 is linked to Rac1 degradation pathway, we investigated the expression level of Caveolin1 in the ovarian samples. We observed a significantly increased level of Caveolin1 in PCOS ovary in comparison to the control (sham) group (Figure 6A and B).

## Discussion

Intra-ovarian signaling stimulates some of the primordial follicles to grow out of a cohort. The quiescent follicles remain in the inactive stage due to inhibitory mechanism operational either within the follicles or by the signals from the ovary [3]. During the post-natal development in mice, a large number of oocytes/follicles are depleted in comparison to the growing population of follicles [42,43]. This phenomenon is similar in the humans as well [3] and depletion of the pool of primodial follicles compromises female fertility. The condition of follicle insufficiency to ovulate is not understood precisely. Previous studies have demonstrated the association of Ras and Rho signaling in the process of ovarian follicle development [43-48]. Rac1 is a member of the Ras family, making it a good candidate for investigation of its role in the regulation of follicular maturation.

The presence of multiple cysts in the ovaries are considered as a key diagnostic trait of PCOS [49-51], which was precisely mimicked in our PCOS mice model. This provided us with a platform for further biochemical and expression analysis. Our histological findings in PCOS ovary correlated well with the earlier reports showing increased ovarian size, absence of ovum and corpus-luteum in the ovaries of DHEA treated mice [52]. A relation between decrease in ovarian volume and the number of follicles with age of women with PCOS has been shown [53]. Increased level of inhibin B and 17β-estradiol in the PCOS group demonstrated elevation in the number of recruited dormant follicles in comparison to the control ovary [36]. Collectively, this confirmed a phenotype of polycystic ovary in our model system.

The estradiol negatively affects Rac1 activation [14], and in turn Rac1 regulates inhibin B [54]; however, this is not known in the ovarian tissue. Rac is a member of small G-protein family (RhoGTPase) and other member of this G-protein family, Ras, has already been implicated in the pathophysiology of ovary [11]. A previous study has suggested involvement of Rac1in gonad formation [12], but its association with follicular maturation and function has not been shown. Our results suggested that expression of Rac1, pRac1 and its activity were significantly reduced in the hyperandrogenized ovary with DHEA (polycystic ovary). Activity of Rac1 was also lowered as compared to sham. It has been reported that higher production of estrogen in vascular smooth muscle cells causes down-regulation of Rac1 [14]. We observed a similar combination of elevated level of intra-ovarian 17β-estradiol and down-regulation of Rac1 on polycystic ovary. Further to confirm the involvement of Rac1, we studied the expression level of total Vav along with its phosphorylated form, which is a known activator of Rac1 [24]. As expected, we observed a reduction in total Vav and its phosphorylated form. The association between Rac1-Vav was further confirmed by immuno-precipitation (IP), which showed Rac1 presence in the immuno-precipitates prepared using anti-Vav from sham treated group. Collectively, all the above results suggest that elevated 17β-estradiol levels might have down-regulated the activity/expression of Rac1 and Vav favoring the development of PCOS phenotype.

Several studies have also shown the interaction of Rac1 with caveolae protein, Caveolin1. Caveolin1 is known to control Rac1 protein levels by regulating ubiquitylation and degradation of activated Rac1 in an adhesion-dependent fashion [55]. The absence of Caveolin1 has been reported to increase the proliferation and anchorage-independent growth by a Rac-dependent, Erk-independent mechanism [56]. Since, there is no evidence that Caveolin1 regulates Rac1 in the ovarian tissue, particularly in PCOS, we analyzed the expression of Caveolin1. A higher level of caveolin1 in PCOS ovary might have signaled a decrease in Rac1 and Vav levels that favors the development of PCOS phenotype. However, this is purely a speculation and further evidence is required to conclude the exact role of Caveolin1 in pathophysiology of PCOS. Rac1 and Caveolin1 are known to associate during cell proliferation signaling [56]; however, Caveolin1 antagonizing function for Rac1 activity in PCOS pathophysiology needs further validation. Herein, our study can infer that Caveolin1 is dysregulated in the PCOS ovary.

On the basis of our observations, we propose that increased androgens levels result in enhanced conversion of estradiol that initiates a series of events leading to the condition of PCOS. It is perhaps increased 17β-estradiol level that results in down regulation of Rac1 and Vav, ultimately, resulting in suspension of follicular development. This leads to arrest of follicular development, and promotes the to accumulation of immature follicles typical to PCOS ovaries. Elevated level of inhibin B is an indicator of repeated recruitment of follicles in the developmental process that is suspended before follicular maturation. How increased 17β-estradiol levels act on Rac/Vav needs to be studied further.

## Conclusion

The results of this study demonstrate for the first time diminished activity of Rac1 and Vav in hyperandrogenized mouse ovaries. Our findings might provide some explanation through small G-protein in the pathogenesis of follicular hyperplasia in PCOS. Altogether, these observations suggest a contribution of elevated estradiol and inhibin B levels due to the DHEA in pathophysiology of PCOS.

## Abbreviations

PCOS: Polycystic ovarian syndrome; DHEA: Dehydroepiandrosterone; CL: Corpus luteum; FC: Follicle cyst; MS: Metabolic syndrome; PVDF: Polyvinylidene fluoride; ECL: Enhanced chemiluminescence; HRP: Horseradish peroxidase; Cat: Catalogue; h: Hour; PFA: Paraformaldehyde; PBS: Phosphate buffer saline; RhoA: Ras homolog gene family member A; Cdc42: Cell division control protein 42 homolog; Rac: Ras-related C3 botulinum toxin substrate; LH: Luteinizing hormone; IP: Immuno-precipitation.

## Competing interests

The authors declare that they have no competing interests.

## Authors’ contributions

VKM carried out PCOS model preparation and development of Rac1, pRac1, Vav and pVav immuno-blot. SC performed Western blotting of Caveolin1, Rac1, pRac1, Vav and pVav along with the 17β-estradiol assay. VK performed the Rac1 activity assay, tissue paraffin embedding and microtomy. SM did the histological examination of ovarian tissue sections and helped in manuscript drafting. AS performed the immuno-precipitation of Vav, immuno-blotting with Rac1 and Vav along with the inhibin B assay. RS assisted in drafting the manuscript and experimental design. RKJ designed, analyzed the data and finalized the manuscript. All authors read and approved the final manuscript.

## Authors’ information

Rajesh Kumar Jha: http://www.cdriindia.org/Rajesh.htm.

Rajender Singh: http://www.cdriindia.org/Rajinder.htm.
